# Heterogeneity in the expression and subcellular localization of POLYOL/MONOSACCHARIDE TRANSPORTER genes in *Lotus japonicus*

**DOI:** 10.1371/journal.pone.0185269

**Published:** 2017-09-20

**Authors:** Lu Tian, Leru Liu, Yehu Yin, Mingchao Huang, Yanbo Chen, Xinlan Xu, Pingzhi Wu, Meiru Li, Guojiang Wu, Huawu Jiang, Yaping Chen

**Affiliations:** 1 Key Laboratory of Plant Resources Conservation and Sustainable Utilization, South China Botanical Garden, Chinese Academy of Sciences, Guangzhou, PR China; 2 Guangdong Provincial Key Laboratory of Applied Botany, South China Botanical Garden, Chinese Academy of Sciences, Guangzhou, PR China; 3 University of Chinese Academy of Sciences, Beijing, PR China; Universidade do Minho, PORTUGAL

## Abstract

Polyols can serve as a means for the translocation of carbon skeletons and energy between source and sink organs as well as being osmoprotective solutes and antioxidants which may be involved in the resistance of some plants to biotic and abiotic stresses. Polyol/Monosaccharide transporter (PLT) proteins previously identified in plants are involved in the loading of polyols into the phloem and are reported to be located in the plasma membrane. The functions of PLT proteins in leguminous plants are not yet clear. In this study, a total of 14 putative *PLT* genes (*LjPLT1*-*14*) were identified in the genome of *Lotus japonicus* and divided into 4 clades based on phylogenetic analysis. Different patterns of expression of *LjPLT* genes in various tissues were validated by qRT-PCR analysis. Four genes (*LjPLT3*, *4*, *11*, and *14*) from clade II were expressed at much higher levels in nodule than in other tissues. Moreover, three of these genes (*LjPLT3*, *4*, and *14*) showed significantly increased expression in roots after inoculation with *Mesorhizobium loti*. Three genes (*LjPLT1*, *3*, and *9*) responded when salinity and/or osmotic stresses were applied to *L*. *japonicus*. Transient expression of GFP-LjPLT fusion constructs in Arabidopsis and *Nicotiana benthamiana* protoplasts indicated that the LjPLT1, LjPLT6 and LjPLT7 proteins are localized to the plasma membrane, but LjPLT2 (clade IV), LjPLT3, 4, 5 (clade II) and LjPLT8 (clade III) proteins possibly reside in the Golgi apparatus. The results suggest that members of the *LjPLT* gene family may be involved in different biological processes, several of which may potentially play roles in nodulation in this nitrogen-fixing legume.

## Introduction

Polyols are a reduced form of aldose and ketose sugars. The carbon chain of polyols can be either linear (acyclic polyols) or cyclic (arranged in a ring). Within the higher plants, at least 13 different polyols have been identified in angiosperms [1]. In some plants, such as woody Rosaceae and celery (*Apium graveolens* L. var. dulce), polyols (mainly sorbitol or mannitol) are, together with sucrose, direct products of photosynthetic carbon fixation. In these species, polyols can perform functions similar to those of sucrose, such as the translocation of carbon skeletons and transfer of energy between sources and sink organs [2–4]. Plant polyols can also function as osmoprotective solutes and antioxidants which may be involved in biotic [5, 6] and abiotic stress tolerance [7–10].

The transport of polyols is performed by Polyol/Monosaccharide Transporter proteins (PLTs or PMTs), which are members of the family of monosaccharide transporters (MSTs). The product of the first polyol transporter gene (*AgMaT1*) isolated from the polyol translocating species celery could function as a mannitol transporter when its cDNA was expressed in *Saccharomyces cerevisiae* [11]. The *AgMaT2* gene product, which was characterized as a mannitol/H^+^ symporter, was shown to be localized to the plasma membrane (PM) of phloem cells [6]. Studies on celery vascular bundles and phloem strands provided evidence in support of an apoplastic step in mannitol phloem loading [3, 12]. The AgMaT1 and AgMaT2 proteins were therefore believed to function in mannitol loading in celery phloem cells. Subsequently, many polyol transporters have been cloned and characterized from a range of polyol-translocating species. In *Prunus cerasus*, *PcSOT1* and *PcSOT2*, which were characterized as encoding sorbitol transporters, were found to be expressed mainly in leaves and fruit (*PcSOT1*) or young fruit (*PcSOT2*) [13]. In *Plantago major*, *PmPLT1* and *PmPLT2* are expressed in the phloem and their products are low-affinity sorbitol/proton symporters [14]. In *Malus domestica*, *MdSOT3*, *MdSOT5* and *MdSOT6*/*MdSOT1* were shown to encode sorbitol/proton cotransporters [15, 16], and the MdSOT6/MdSOT1 protein was localized to the PM and interacted with cytochrome b5 to regulate its affinity for substrate sugars [16]. In *Olea europaea*, mannitol transport and mannitol dehydrogenase activities are coordinated under salinity and osmotic stresses. Salinity and drought stresses significantly increased mannitol transport activity and induced *OeMaT1* expression in *O*. *europaea* cell suspensions [8].

Non-polyol-translocating plant species also have PLT-type genes. In Arabidopsis, six putative PLT transporters have been isolated, but only the AtPLT1, AtPLT2 and AtPLT5 proteins, which are confined to the PM, have been the subjects of further study. *AtPLT5* has been characterized as a broad-spectrum H^+^-symporter for linear polyols, cyclic polyol myo-inositol and several different hexoses and pentoses [17, 18]. However, *AtPLT1* and *AtPLT2*, which were identified as xylitol and fructose transporters, were found to be expressed in developing xylem and pollen [19]. Other PLT proteins previously reported in non-polyol-translocating plants are all located on the PM; examples include *HbPLT2* from *Hevea brasiliensis* and *VvPLT1* from *Vitis vinifera*. Yeast expressing *HbPLT2* displayed active absorption of xylitol but a marginal level of absorption of inositol, mannitol and sorbitol [20]. Moreover, in these yeast cells, xylitol uptake was reduced by increasing concentrations of quebrachitol, which is a cyclic polyol and is one of the main sugars in *H*. *brasiliensis*. It was therefore suggested that *HbPLT2* is a quebrachitol transporter [20]. *VvPLT1* is also an H^+^-dependent polyol transporter, which has high affinities for sorbitol and mannitol [9]. The expression level of the *VvPLT1* gene was upregulated in response to salt and water-deficit stresses, as well as by exogenous abscisic acid (ABA) and salicylic acid treatments [9].

In *L*. *japonicus*, at least 10 polyols have been detected in seedlings and nodules [21, 22]. A putative PLT protein (Acc. No. in NCBI: BI420529; Lj3g3v3338420.1) identified at the peribacteroid membrane of *L*. *japonicu*s indicates that polyols can be exchanged between the plant and bacteroids in nitrogen-fixing legume nodules [23]. The massive accumulation of sugar alcohols and the high expression level of sorbitol dehydrogenase in nodules offer clear evidence that polyol biosynthesis occurs in nodules [21]. Four putative *PLT* genes have been isolated from *L*. *japonicus*, and the *LjPLT4* (*Lj3g3v3338420*.*1*) gene was found to encode a PM xylitol-specific H^+^-symporter [24]. In this study, we identified a total of 14 genes putatively encoding PLT transporter proteins in the *L*. *japonicus* genome. Phylogenetic analysis indicated that the *LjPLT* genes are divided into 4 clades. The transcription patterns of the gene family in different tissues and in response to abiotic and biotic stresses were analyzed by quantitative real-time PCR (qRT-PCR). The subcellular localization of the proteins was studied with GFP (green fluorescent protein)-LjPLT fusions and the results showed that some of the LjPLTs are likely to be mainly localized on the Golgi apparatus.

## Materials and methods

### Plant growth conditions and treatments

*L*. *japonicus* MG-20 was used in this study. For seed germination, seed coats were treated with concentrated sulfuric acid for 5 min and washed six times with sterile water. Next, seeds were surface sterilized with 2% sodium hypochlorite solution for 10 min and put into square Petri dishes for 3 d. The germinated seeds were transferred to square Petri dishes with 1/2-strength Broughton & Dilworth medium (1/2 B&D) [25] and a piece of wet sterile filter paper. The plants were grown in an artificially lit growth cabinet at 22°C for 16 h (light) and 18°C for 8 h (dark) with a constant relative humidity of 70%.

For expression analysis, MG-20 seedlings were planted on plates with 1/2 B&D. In order to investigate whether *LjPLT* genes are involved in the *Rhizobium*-legume symbiosis, 10-day-old seedlings were inoculated with *M*. *loti* MAFF303099100, a kind of nitrogen-fixing rhizobium that has symbiotic relationship with *L*. *japonicus*. Whole roots were harvested at 0, 1, 2, 3, and 7 days after infection. For salt and osmotic stresses response analysis, MG-20 seedlings were planted in sterilized pots containing vermiculite with 1/4 B&D and 5 mM KNO_3_. After 10 days, seedlings were irrigated with 10% PEG or 100 mΜ NaCl, and whole roots and shoots were harvested at 6, 12, 24 and 48 h after treatments. In all cases, three independent replicates were used in each of three biological experiments and data were analyzed with a Duncan test [26] using the SAS software package (http://www.sas.com/en_us/software/sas9.html).

### Sequence database searches

Sequences of Arabidopsis PLT proteins were downloaded from the Arabidopsis genome database, TAIR (http://www.Arabidopsis.org/), sequences of *Solanum lycopersicum*, *Glycine max*, *Phaseolus vulgaris*, *Oryza sativa*, and *Sorghum bicolor* PLT proteins were obtained from Phytozome (http://phytozome.jgi.doe.gov/pz/portal.html), and other PLT protein sequences were downloaded from GenBank (http://www.ncbi.nlm.nih.gov/). We searched for PLT genes in the *L*. *japonicus* genome database of the Kazusa DNA Research Institute (http://www.kazusa.or.jp/lotus/). We used Arabidopsis PLT proteins as query sequences for Blastp and Blastn searches against the *L*. *japonicus* genome sequences and against predicted protein sequences. Next, we corrected errors in the annotation of *PLT* coding domain sequences according to the cDNA clones and their sequences.

### Phylogenetic tree construction

Multiple sequence alignments of putative full-length PLT amino acid sequences were performed using MUSCLE (http://www.ebi.ac.uk/Tools/msa/muscle/) [27]. Unrooted trees were constructed using the neighbor-joining (NJ) method and 1000 bootstraps and the results were displayed with the MEGA software package version 5.0 [28].

### In silico analysis

The promoter analysis was done by retrieving upstream 2 kb sequences of the fourteen *LjPLT* genes. The *cis-*regulatory elements in the promoter analysis were analyzed in PLANTCARE database (http://bioinformatics.psb.ugent.be/webtools/plantcare/html/).

### RNA isolation and expression analysis

Total RNA was isolated from tissues of *L*. *japonicus* using a HiPure Plant Mini Kit (Magen; http://www.magentec.com.cn/) according to the manufacturer’s instructions. After treating the RNA with DNase I and purifying it with a Magen RNeasy column, first-strand cDNAs were synthesized from 2 μg RNA using a GoScript^TM^ Reverse Transcription System (Promega; http://cn.promega.com/) according to the manufacturer’s instructions.

Quantitative real-time analysis was performed using a *L*. *japonicus* adenyl pyrophosphatase (*LjATPase*) gene and a *LjUBC* gene (encoding ubiquitin-conjugating enzyme) as internal controls; the primer sets are listed in S1 Table. A Mini Option real-time PCR system (LightCycler 480: Roche, http://www.roche.com/) was used for all qRT-PCR assays, and Promega Go Taq qPCR Master Mix (http://cn.promega.com/) was used according to the manufacturers’ instructions. Cycling conditions were as follows: 10 min at 95°C for DNA polymerase activation, followed by 40 cycles of 5 s at 95°C, 20 s at 60°C and 20 s at 72°C. Expression levels were calculated using the 2^-ΔΔCT^ method. Each PCR assay was run in triplicate for each of three independent biological repeats.

### Cloning of *LjPLT* genes and vector construction

For subcellular location analysis, complete coding sequences without stop codons were amplified by RT-PCR using the primers listed in S1 Table. Modified coding sequences of *LjPLTs* for localization studies were cloned into the corresponding site in the vector pUC18-GFP, which carried an in-frame GFP fusion at its 5’-end. The cloned sequences were digested with Kpn I and BamH I and used as described in the In-Fusion® HD Cloning Kit User Manual. The plasmid constructs were then delivered into Arabidopsis protoplasts. For co-localization, we used tobacco (*N*. *benthamiana*) containing a red fluorescent protein tagged Golgi marker. The plants were grown on half-strength MS medium in a growth chamber under the following conditions: 22°C for 16 h (light) and 18°C for 8 h (dark). After 8 weeks, protoplasts were extracted from the tobacco and 10 μg plasmid was transformed into the protoplasts using the PEG transfection method [29].

### Laser scanning confocal microscopy

For subcellular localization of the GFP-LjPLT fusion proteins in Arabidopsis protoplasts, fluorescence images were analyzed with a Leica TCS SP8 confocal microscope using a 63 × 1.40 oil objective. Images were captured at 488 nm and 552 nm laser excitation, and using 495 to 550 nm and 650 to 750 nm long-pass emission filters for GFP and chlorophyll respectively.

For co-subcellular localization of the GFP-LjPLT proteins in protoplasts of *N*. *benthamiana*, GFP-LjPLT fusion constructs were transiently expressed in *N*. *benthamiana* expressing a marker of the Golgi apparatus, mCherry-tagged alpha-mannosidase II (AMAN-2). A confocal laser microscope (Leica TCS SP8X) equipped with a hybrid detector and a highly flexible pulsed white-light laser was used. The Leica Application Suite X (LAS X) was used as a software platform, and the objective lens used was the 63 × 1.40 oil. Green fluorescent protein (GFP) was measured with excitation at 488 nm from the white-light laser, and with emission at 495 to 550 nm via the HyD detector. The emission spectra of mCherry were measured with excitation at 561 nm from the white light laser, and with emission at 570 to 630 nm via the HyD detector. To capture autofluorescence images, the white light laser was used with excitation at 488 nm, and the PMT detector was set to an emission range of 650 to 750 nm.

The 3-D images were acquired using a Nikon A1 confocal microscope and a 60× oil objective. Images were captured at 488 nm, 640 nm and 561 nm laser excitation and with 500 to 550 nm, 675 to 725 nm and 570 to 620 nm emission filters for GFP, chlorophyll and mCherry respectively. Three-dimensional reconstructions were accomplished using the Nikon Elements software package (Nikon Inc., Melville, NY).

## Results

### Identification of *PLT* genes in *L*. *japonicus*

**Fig 1:**
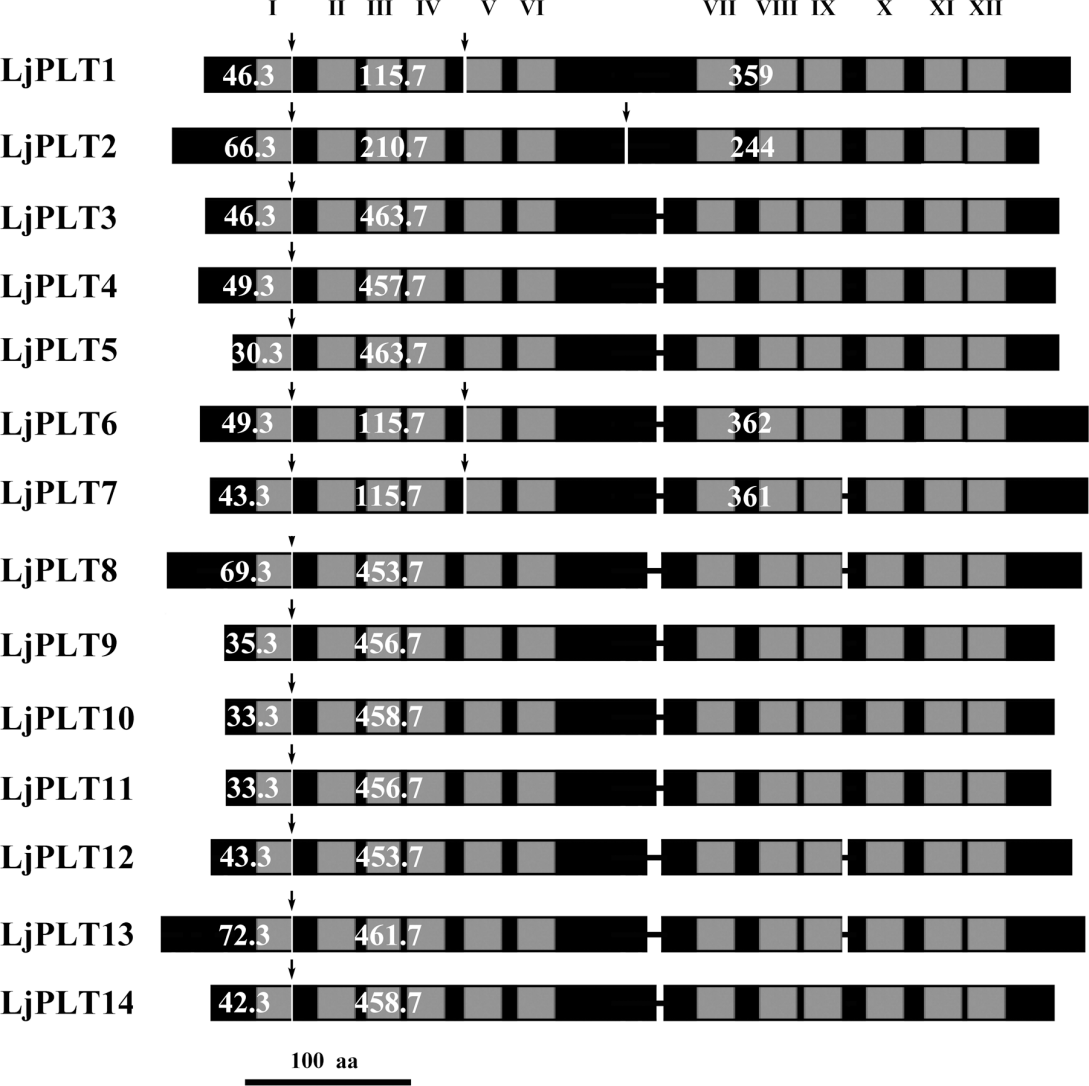
Comparison of the 14 LjPLT proteins. Schematic alignment of the deduced protein sequences (boxes) of LjPLT1 to LjPLT14 based on the positions of intron (arrows) in these genes. Grey boxes (I–XII) indicate the positions of transmembrane helices as predicted by the HMMTOP software package. Thin lines show small gaps in the sequences. Numbers of amino acids encoded by the different exons are indicated (white).

BLAST searches of the *L*. *japonicus* genome proteome assembly build 2.5 and 3.0 database, using the amino acid sequences of 6 Arabidopsis PLTs as queries, enabled us to identify 17 loci putatively encoding *PLTs*. In the *L*. *japonicus* genome assembly build 3 database, the gene pair *Lj2g3v1758430*.*1* and *Lj2g3v1778720*.*1* shared a very high degree (>99%) of nucleotide sequence identity as did the gene pair *Lj2g3v1758440*.*1* and *Lj2g3v1778740*.*1*; however, only one gene from each of these pairs was identified in the build 2.5 database. This may be the result of a technical or human error during the assembly of the genomic sequences. Here, we used only the *Lj2g3v1758430*.*1* and *Lj2g3v1758440*.*1* genes in further analysis. *Lj4g3v3071660*.*1*—*Lj4g3v3071670*.*1* did not contain complete coding domain sequences for a PLT protein. The 14 genes putatively encoding full-length PLT proteins were named *LjPLT1* to *LjPLT14* according to their loci on the *L*. *japonicus* chromosomes/scaffolds. Among these genes, four encode previously described *PLTs* [24]. Tandem duplications, defined as tandem repeats which were located within 50 kb from each other or were separated by < 4 non-homologous spacer genes [30], were observed for the *PLT* genes in the *L*. *japonicus* genome. Three genes (*LjPLT3*, *4*, *5*) on chromosome 2 and three genes (*LjPLT9*, *10*, *11*) on chromosome 4 are located in tandem repeat regions. Analysis of exon/intron structures showed that 4 *LjPLT* genes have two introns, while the other 10 *LjPLT* genes have single intron in their open reading frames (Fig 1). Detailed information about these *LjPLT* genes is listed in S2 Table.

Most monosaccharide transporters have 12 membrane-spanning domains separated by a cytoplasmic loop between transmembrane helices 6 and 7 [31]. The deduced amino acid sequences of the 14 *LjPLT* genes identified here appeared to have 12 putative membrane-spanning domains with 6 + 6 models (Fig 1) based on hydropathy analysis using the HMMTOP 2.1 software package [32] for the prediction of transmembrane segments in a protein (http://www.sacs.ucsf.edu/cgi-bin/hmmtop.py) and Phobius [33] for the prediction of transmembrane topology and signal peptides (http://phobius.sbc.su.se/).

**Fig 2:**
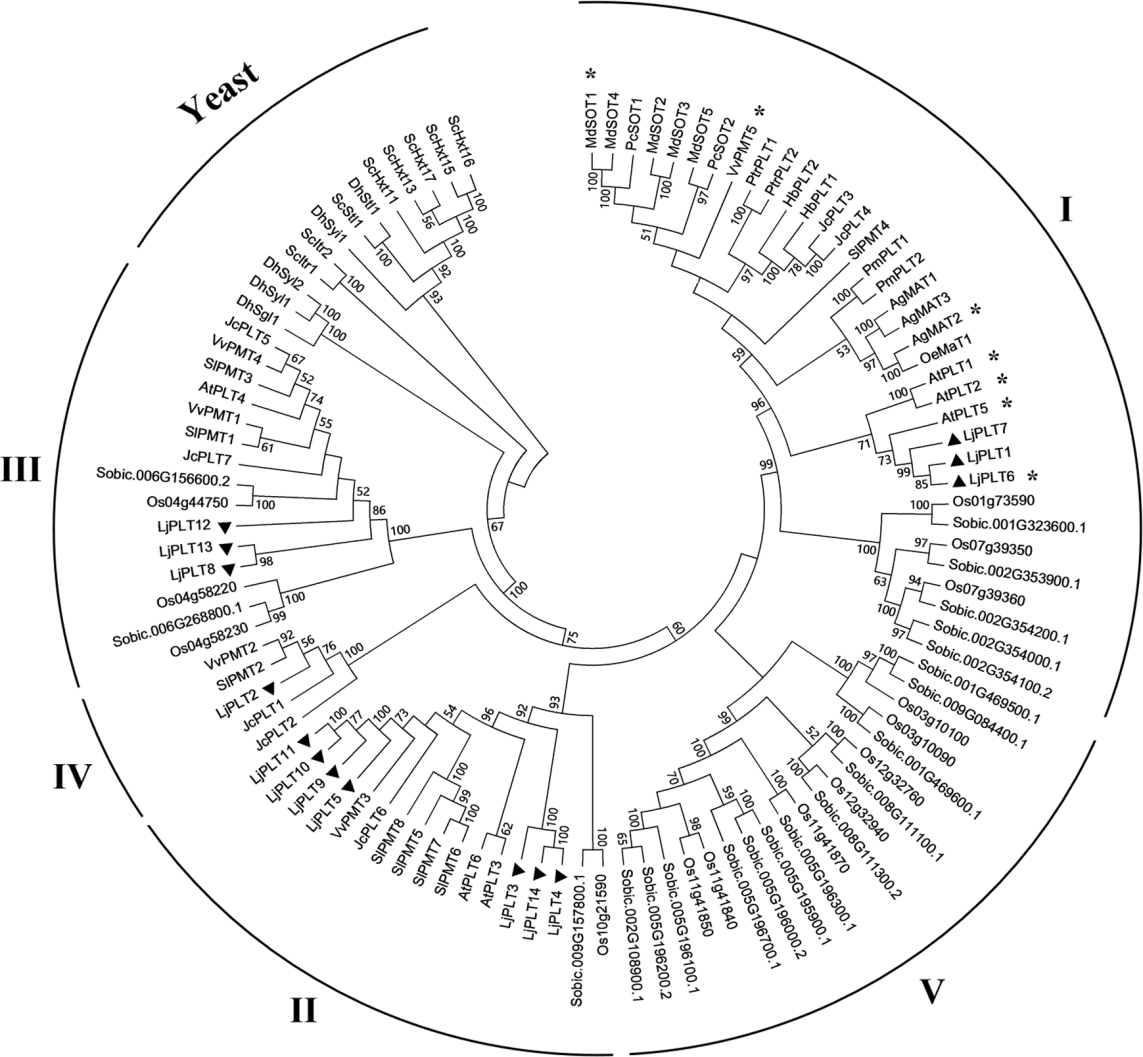
Phylogenetic tree of polyol transporters in different species. The tree was constructed using the neighbor-joining method with 1000 bootstrap replications. Asterisks indicate proteins of plant that have been reported to be localized to the plasma membrane. Accession numbers for the *PLT* genes are listed in S3 Table.

We inferred the phylogenetic relationships of the LjPLT proteins with proteins from Arabidopsis, *S*. *lycopersicum*, *Jatropha curcas*, *V*. *vinifera*, *O*. *sativa*, *S*. *bicolor*, *Saccharomyces cerevisiae*, and *Debaryomyces hansenii* [34, 35], and functionally analyzed PLT proteins from several plants (Fig 2), as well as proteins from *G*. *max* and *P*. *vulgaris* (S1 Fig), using neighbor-joining within the program MEGA 5.0. The phylogenetic tree showed that the PLT family had diverged into 6 clades (Fig 2). Clade IV and clade V contain PLT proteins from only dicots and only monocots, respectively. PLT proteins of yeast was divided into an independent clade. No PLT protein from Arabidopsis fell into clade IV. Most (7) of the *L*. *japonicus* genes were in clade II, which was divided into two subclades, IIa (3) and IIb (4). Three genes, *LjPLT3*, *4*, and *5*, which were located in a tandem repeat region on chromosome 2, fell into subclade IIa (*LjPLT3* and *4*) and subclade IIb (*LjPLT5*) (Fig 2). Tandem gene duplication and divergence of clade II *PLTs* were also observed in the genomes of *G*. *max* and *P*. *vulgaris*. Subclade IIa genes of *Phvul*.*002G028200*.*1* and *Phvul*.*002G028300*.*1*, and subclade IIb genes of *Phvul*.*002G028500*.*1* and *Phvul*.*002G028600*.*1*, in *P*. *vulgaris*, and subclade IIa genes of *Glyma*.*11G066000*.*1*, *Glyma*.*11G066100*.*1*, *Glyma*.*11G066300*.*1*, and *Glyma*.*11G066400*.*1*, and subclade IIb genes of *Glyma*.*11G066500*.*1* and *Glyma*.*11G066600*.*1*, in *G*. *max*, were located in tandem repeat regions (S1 Fig and S3 Table). In legumes, proteins in both clade I and clade III could be divided into three subclades (S1 Fig). All of the plant PLT proteins which have been previously reported to function as transporters for polyols and/or monosaccharides, and to be localized to PM, fell into clade I. *L*. *japonicus* have three proteins (LjPLT1, 6, 7) in clade I; of these, LjPLT6 was characterized as a PM xylitol H^+^-symporter, as was LjPLT4 in a previous study [24].

**Fig 3:**
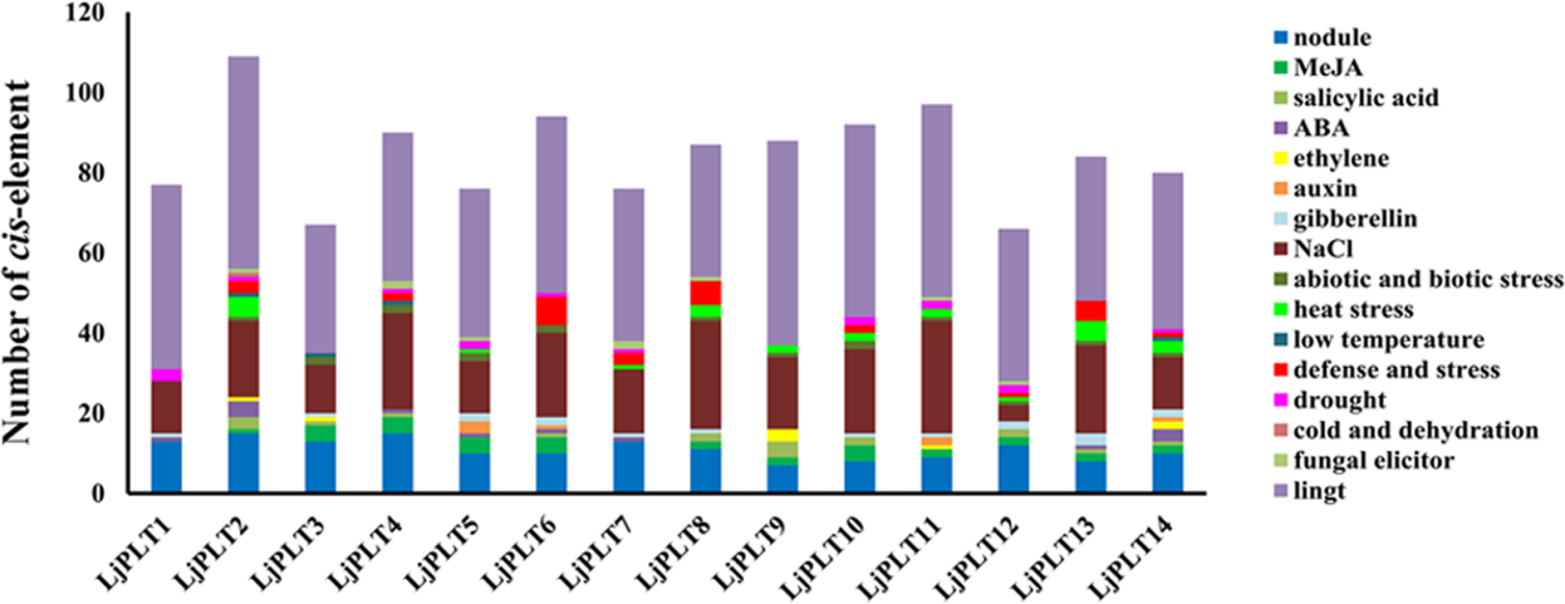
*Cis*-element analysis of putative *LjPLT* promoters related to stress responses. Different *cis*-elements with the same or similar functions are shown in the same color.

Expression of genes is regulated by the specific *cis*-regulatory elements present in promoter sequences. Upstream sequences of 2 kb from the predicted start codon of the *LjPLTs* were retrieved and analyzed in PLANTCARE database for the identification of putative *cis*-regulatory elements. As shown in Fig 3 and S4 Table, light-responsive elements accounted for the majority of elements in every *LjPLT* promoter. There were 20 to 36 different types of light-responsive elements present in the 14 *LjPLT* promoters. This result indicated that *LjPLTs* might be differentially regulated when subjected to light. Different kinds of hormone-responsive regulatory elements were found in the *LjPLT* promoters, such as *cis*-acting regulatory element involved in the MeJA, SA, ABA, ethylene, gibberellins and auxin responsiveness. Moreover, 10 types of stress-responsive regulatory elements, GT1GMSCAM4, ASF1MOTIFCAMV, HSE, LTR, TC-rich repeats, MBS, C-repeat/DRE, ARE, Box-W1 and WUN-motif, with responses to NaCl-induction, Abiotic and biotic stress, heat stress, low-temperature, defense and stresses, drought inducibility, cold stress, anaerobic induction, fungal elicitors and wound induction, respectively, were identified in the *LjPLT* promoters. Different types and numbers of regulatory elements were present in variant *LjPLT* promoters, indicating that *LjPLT* genes should have different regulatory mechanisms in response to various stress and hormone treatments. Different type of nodule-specific sequences was present in the *LjPLT* promoters, which suggested that *LjPLT* gene could play roles in nodulation in legume plants.

### Expression of *LjPLT* genes in different tissues

**Fig 4:**
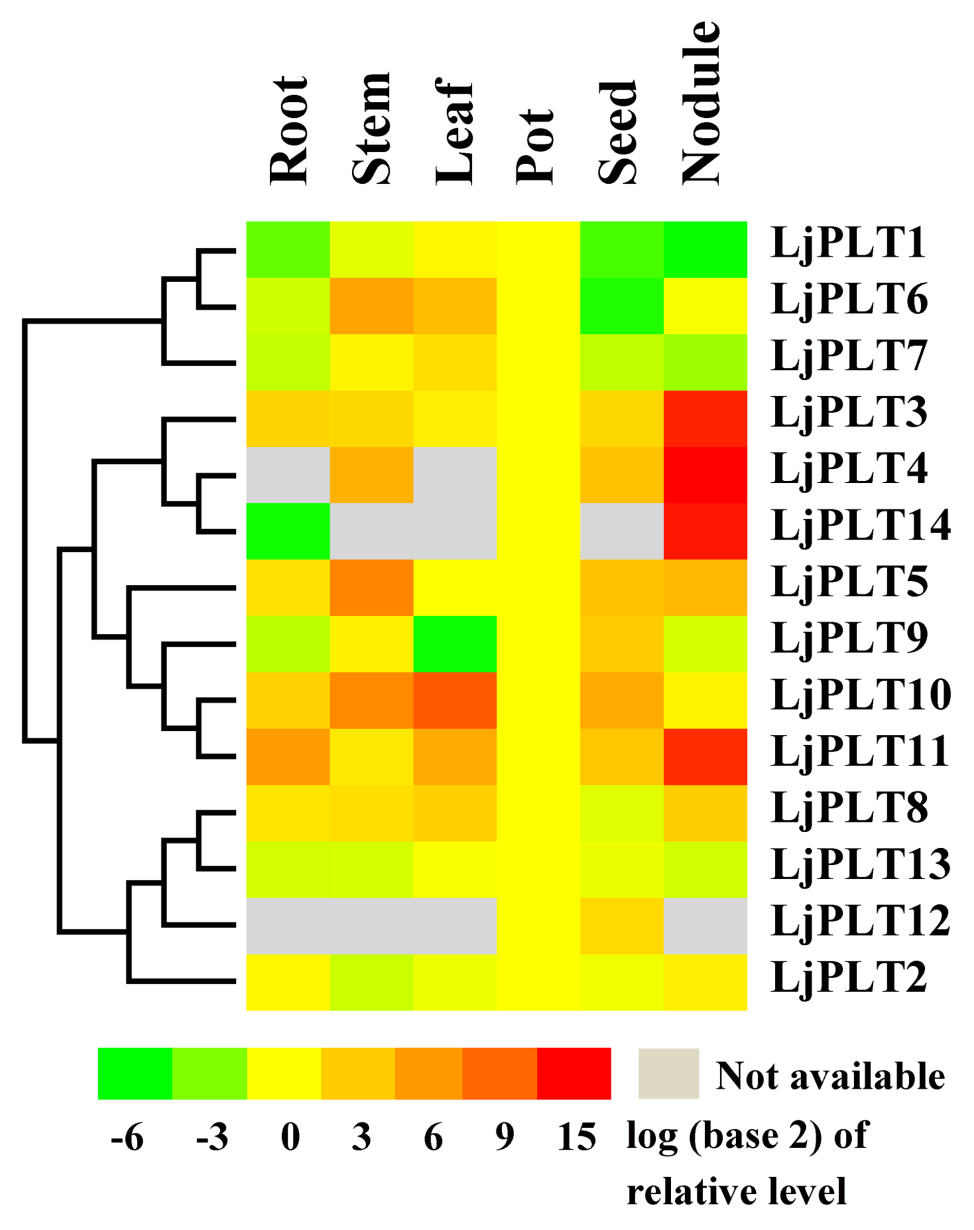
Expression patterns of *LjPLT* genes. The relative expression levels of the *LjPLTs* in different organs were tested by qRT-PCR. Relative expression was normalized to the reference genes *LjATPase* and *LjUBC* (internal control) and the expression level in pods was defined as “1”.

The expression levels of *LjPLT* transcripts were measured by qRT-PCR using leaves, roots, stems and nodules from 3-week-old plants, and young pods and seeds from 10-to 12-week-old plants. The results showed that most of the *LjPLT* genes were expressed in differing abundances in the tissues tested (Fig 4). Three genes (*LjPLT1*, *6*, *7*) of clade I were expressed more highly in chlorenchyma, stems, leaves and young pods than in other tissues. For the clade III genes, *LjPLT12* was expressed mainly in pods and seeds, whereas *LjPLT8* was weakly expressed in these tissues. Three genes (*LjPLT4*, *5*, *10*) in stems, two genes (*LjPLT10*, *11*) in leaves, one gene (*LjPLT11*) in roots, and four genes (*LjPLT4*, *5*, *9*, *10*) in seeds were expressed at relatively high levels.

Since *L*. *japonicus* is a nitrogen-fixing legume species, we tried to find genes that might play roles in nodulation. We found that four genes (*LjPLT3*, *4*, *11*, *14*) from clade II were expressed at a significantly higher level in nodules compared to other tissues tested, and it was likely that *LjPLT14* was expressed only in nodules. In particular, the genes *LjPLT3*, *4*, *5*, *11* and *14* were transcribed at much (more than 3-fold) higher levels in nodules than in roots, and *LjPLT4* and *LjPLT14* were barely expressed in roots (Fig 4).

### Response of *LjPLT* genes to *M*. *loti* in *L*. *japonicus* roots

**Fig 5:**
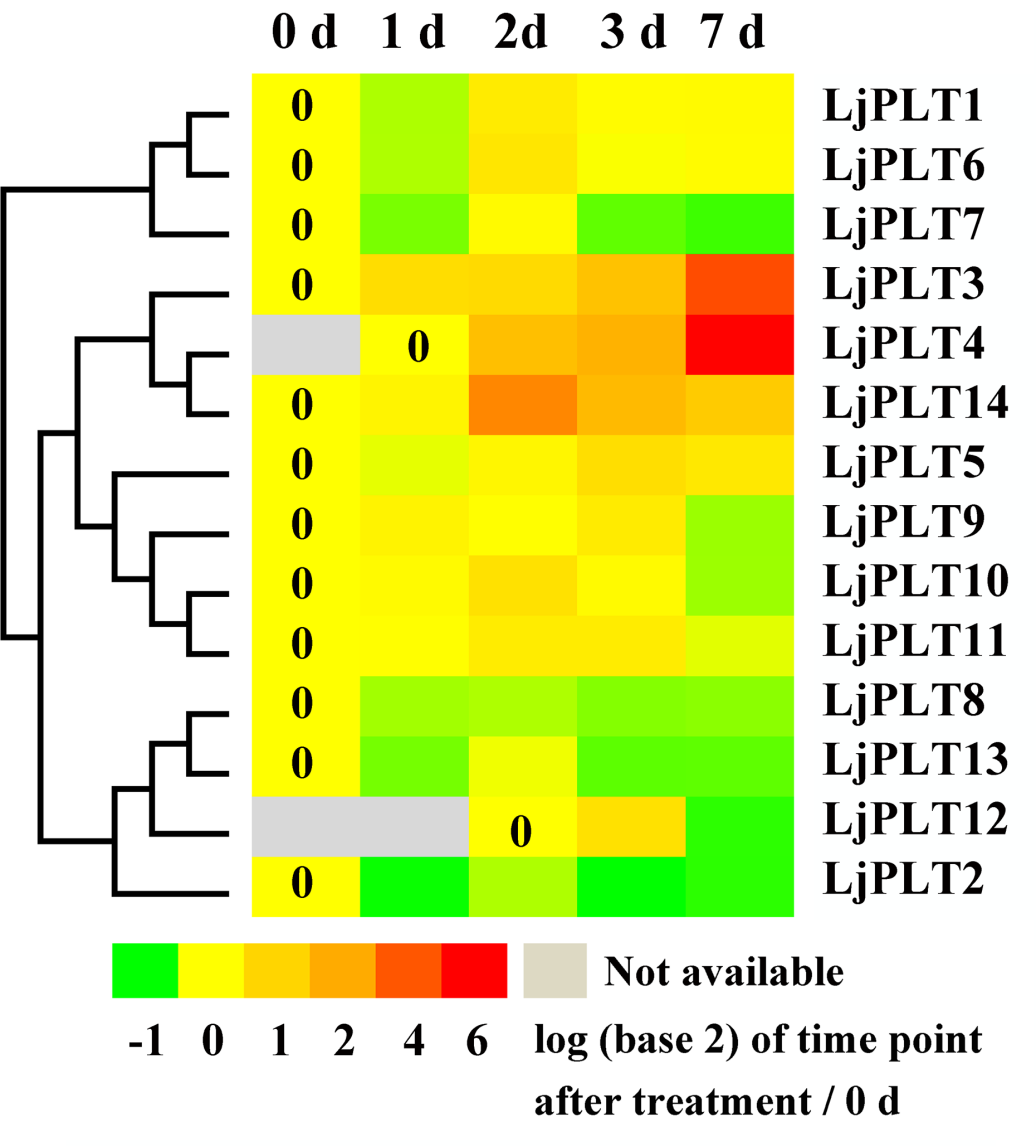
Expression patterns of *LjPLT* genes in response to *M*. loti. Levels of *LjPLT* transcripts in infected roots at 0, 1, 2, 3 and 7 d after inoculation with *M*. *loti*.

In *L*. *japonicus* roots and nodules, at least 10 polyols have been detected and polyol biosynthesis has also been reported to occur [21, 22, 36]. These results suggest that polyol transporters may be involved in the nitrogen-fixing *Rhizobium*-legume symbiosis. Here we tested the expression levels of *LjPLT* genes in *L*. *japonicus* roots in response to *M*. *loti* inoculation at different time points. Although *LjPLT3*, *LjPLT4*, *LjPLT11* and *LjPLT14* are all expressed strongly in nodules (Fig 4), only the clade II genes (*LjPLT3*, *4*, and *14*) showed a significant increase in expression in roots after inoculation (Fig 5). The expression levels of *LjPLT3* and *LjPLT4* increased steadily in roots after inoculation, while the expression of *LjPLT14* reached its highest level at the two day point and subsequently decreased (Fig 5). The expression of *LjPLT2* decreased about 2-fold in roots by time points 1 d, 3 d, and 7 d after inoculation (Fig 5).

### Response of *LjPLT* genes to salinity and osmotic stresses in *L*. *japonicus*

**Fig 6:**
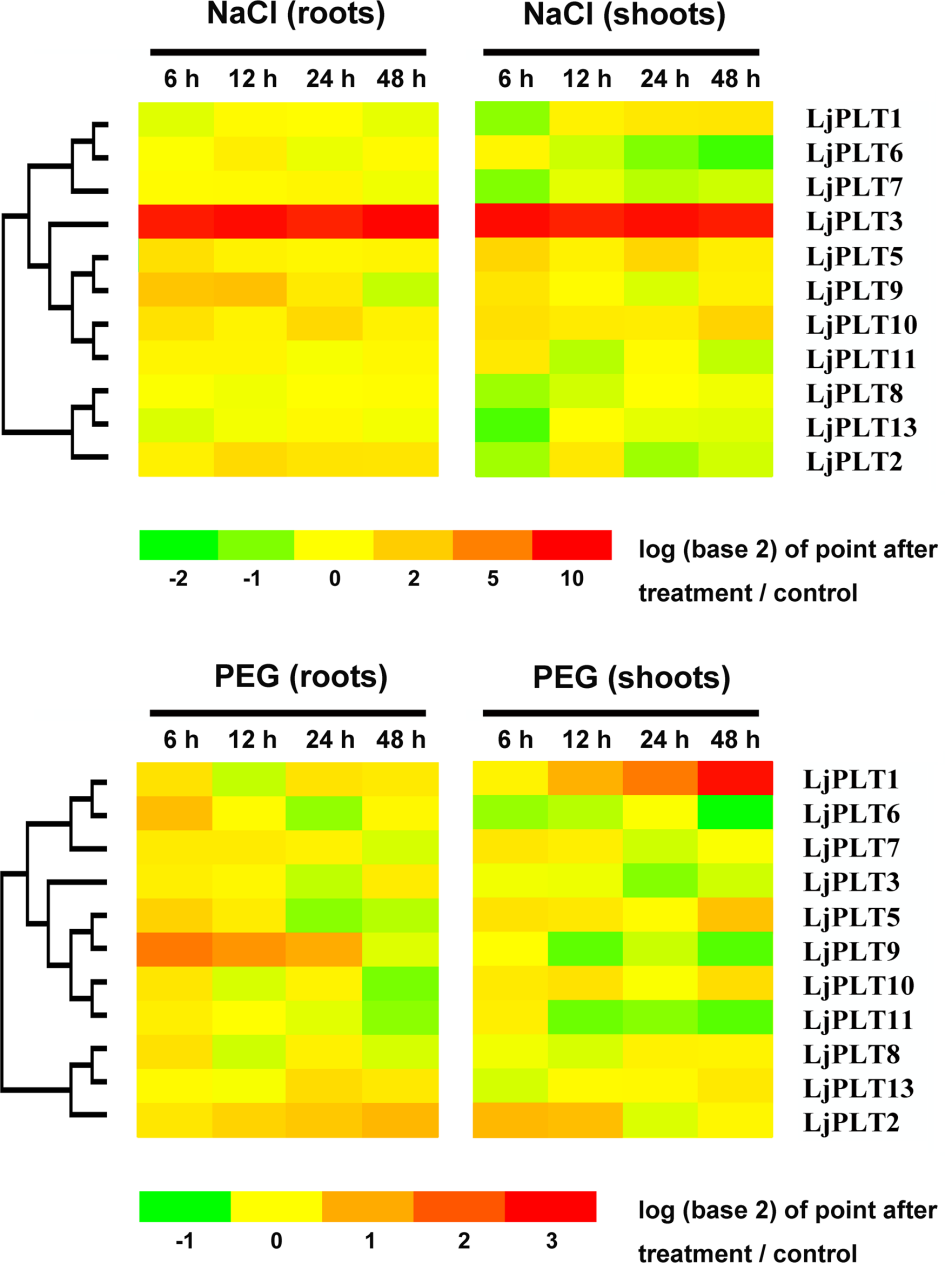
The response of *LjPLT* genes to salt and osmotic stresses applied to *L*. *japonicus*. Expression of *LjPLTs* in roots and shoots at different time points after 10% PEG and 100 mM NaCl treatments.

Polyols in plants play roles in resistance to abiotic stresses. The up-regulation of *PLT* genes expression levels were analyzed by qRT-PCR under salinity and water-deficiency stresses has been reported for several plants [8, 9]. In order to investigate whether *LjPLT* genes are responsive to salinity and osmotic stresses, their expression levels in roots and shoots was tested by qRT-PCR after treatment with 10% PEG or 100 mM NaCl. As shown in Fig 6, the expression of *LjPLT1* (clade I) was up-regulated in shoots but not in roots under both salinity and osmotic stresses, and its expression level increased gradually during the treatments. The expression level of *LjPLT3* (clade II) increased over 400-fold in both roots and shoots at points 6 h to 48 h after the salinity stress treatment (Fig 6). The expression of *LjPLT9* (clade II) increased in roots at points 6 h and 12 h after the salinity and osmotic stress treatments.

### Subcellular localization of the LjPLT proteins

Sugar transporters may be localized to, and function at, the PM, chloroplasts, vacuoles or Golgi apparatus [37, 38]. In previous reports, all of the PLT proteins tested from different plant species were shown to be localized to the PM. To further investigate the subcellular localization of LjPLTs, transient expression of GFP-LjPLT constructs in Arabidopsis and *N*. *benthamiana* was conducted. Protoplasts of Arabidopsis and/or *N*. *benthamiana* expressing markers for Golgi apparatus, mitochondrion and endoplasmic reticulum tagged with fluorescent proteins were used.

**Fig 7:**
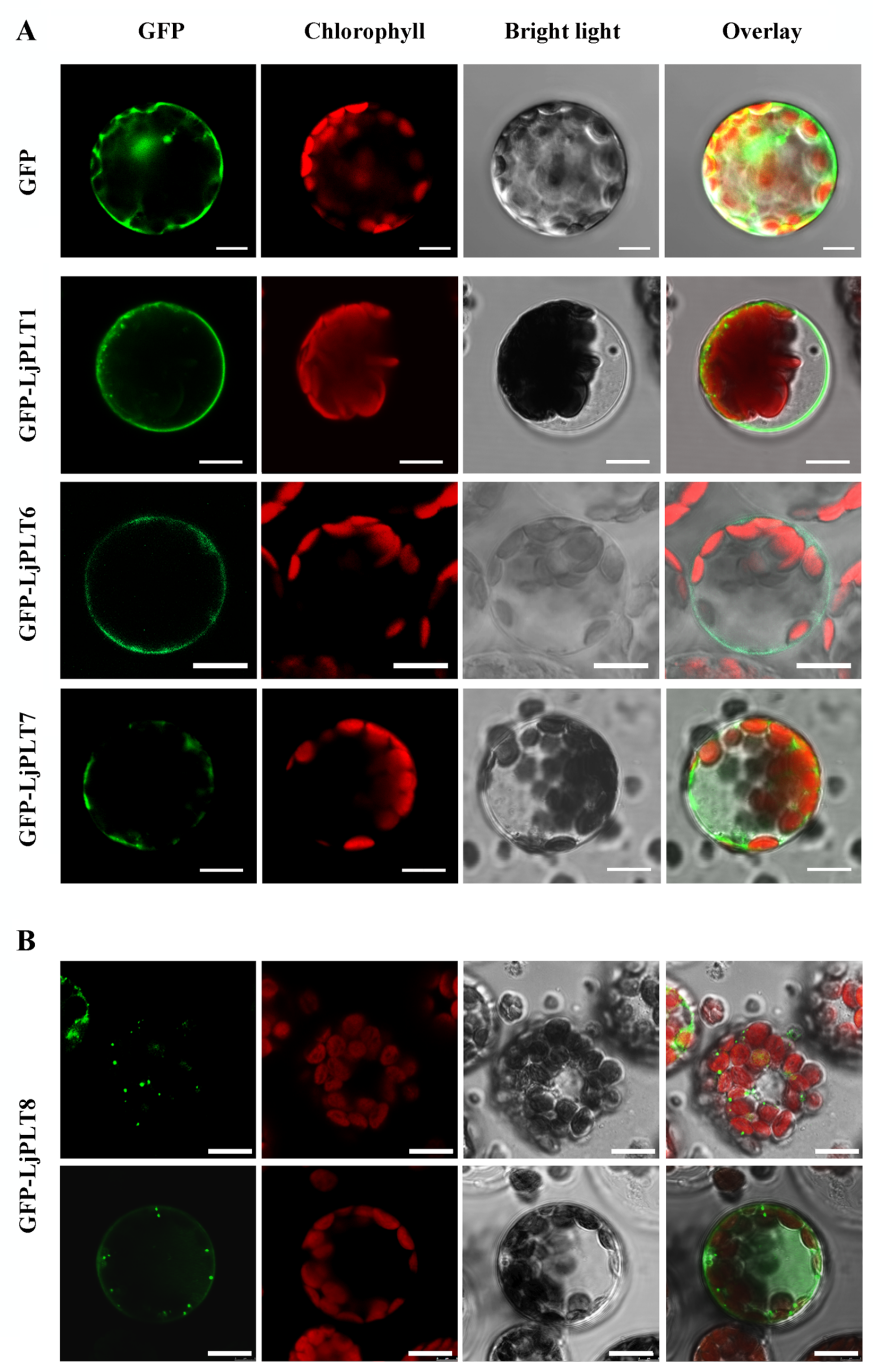
Transient expression of GFP-LjPLT fusion constructs in Arabidopsis protoplasts. (A) GFP-LjPLT1, GFP-LjPLT6 and GFP-LjPLT7 are localized in the plasma membrane. Scale bar, 5 μm. (B) GFP-LjPLT8 is localized both in the cytoplasm and at the plasma membrane. The images were recorded with a Leica TCS SP8 confocal microscope. Scale bar, 5 μm.

**Fig 8:**
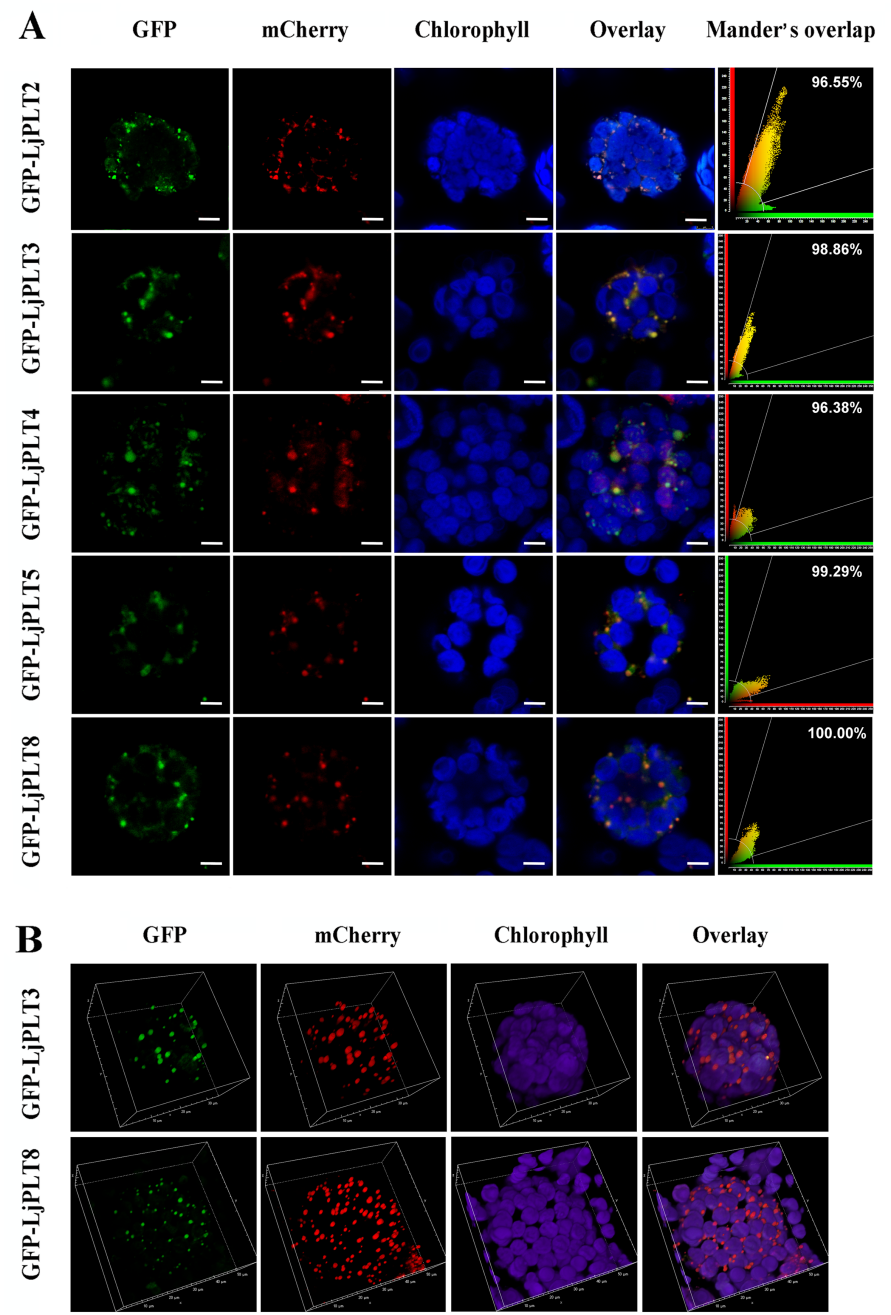
Transient expression of GFP-LjPLT fusion constructs in *N*. *benthamiana* expressing a marker of the Golgi apparatus, mCherry-tagged alpha-mannosidase II (AMAN-2). (A) The proteins GFP-LjPLT2, GFP-LjPLT3, GFP-LjPLT4, GFP-LjPLT5 and GFP-LjPLT8 co-localized with mCherry-tagged AMAN-2. The images were recorded with a Leica TCS SP8 X confocal microscope, in the GFP region with time gating (gate on time: 0.0–12.0 ns) and in the mCherry region with time gating (gate on time: 0.3–11.9 ns). Scale bar, 5 μm. (B) GFP-LjPLT3 and GFP-LjPLT8 are localized in the Golgi apparatus as shown by 3-D reconstructions. 3-D Images were acquired using a Nikon A1 confocal microscope, with a 60× oil objective.

Firstly, we transiently expressed the three recombinants proteins GFP-LjPLT1, GFP-LjPLT6 and GFP-LjPLT7 (clade I) in Arabidopsis protoplasts. Confocal microscopy showed GFP fluorescence in both the cytoplasm and nucleus in control GFP-expressing cells (Fig 7A). In contrast, in cells expressing GFP-LjPLT1, GFP-LjPLT6 and GFP-LjPLT7, the green fluorescence was confined to the PM (Fig 7A). Next, we transiently expressed the other five recombinant proteins, GFP-LjPLT8 (clade III), GFP-LjPLT3, GFP-LjPLT4, GFP-LjPLT5 (clade II) and GFP-LjPLT2 (clade IV), in Arabidopsis protoplasts. In most (ca. 80%) of the GFP-LjPLT8 expressing cells, the green fluorescence was localized only in the cytoplasm, appearing as strongly fluorescent spots, while in a few cells (ca. 20%) the fluorescence was localized both to the cytoplasm and to the PM (Fig 7B). In GFP-LjPLT3, GFP-LjPLT4, GFP-LjPLT5 and GFP-LjPLT2 expressing cells, the green fluorescence was similarly mainly localized in the cytoplasm (data not shown). To determine in which organelles these LjPLT proteins were located, we expressed the recombinant proteins in protoplasts of *N*. *benthamiana* containing Golgi apparatus, mitochondrion, or endoplasmic reticulum tagged with a red fluorescent protein fusion marker gene. When the GFP-LjPLT constructs were transformed into protoplast cells expressing a marker for the Golgi apparatus, most of the green fluorescence arising from GFP-LjPLT could be merged with the red fluorescence (Fig 8A). GFP-LjPLT3 and GFP-LjPLT8 expressing cells were selected for viewing of their 3-D reconstructions. Complete mixtures of the red and green fluorophores were seen, suggesting that the signals fully overlapped (Fig 8B).

## Discussion

In the present study, a total of 14 *PLT* genes were identified in the *L*. *japonicus* genome. The LjPLT proteins could be divided into 4 clades according to the results of phylogenetic analysis (Fig 2 and S1 Fig). Hydropathy analyses of the protein sequences showed that LjPLTs have 6 + 6 transmembrane domains (Fig 1). Gene duplication has occurred throughout plant evolution, contributing to the establishment of new gene functions and underlying the origins of evolutionary novelty [30, 39]. In apple, *MdSOT3*, *MdSOT4*, and *MdSOT5* from source leaves are more closely related to polyol transporter homologs from Rosaceae than to those from other families. This suggests that the Rosaceae sorbitol transporter homologs diverged after the Rosaceae had evolved away from other families [15]. Similarly, in *L*. *japonicus* three *PLT* genes in clade I and three in clade III may have originated from gene duplication events and diverged after the *Leguminosae* had evolved from other families. Most of the *L*. *japonicus PLT* genes fell into clade II (Fig 2 and S1 Fig). Two tandem arrays of clade II genes were identified (S2 Table), one of which was also detected in the genomes of other *Leguminosae* species such as *G*. *max* and *P*. *vulgaris* (S1 Fig). *LjPLT3*, *4*, and *5*, which are located in a tandem repeat region on chromosome 2, fell into subclade IIa (*LjPLT3* and *4*) and subclade IIb (*LjPLT5*) (S1 Fig). This tandem duplication and divergence of genes were also observed in the genomes of *G*. *max* and *P*. *vulgaris* (S1 Fig and S3 Table). These results suggested that the expansion of the *PLT* gene family in *L*. *japonicus* resulted from duplication events that occurred both before and after the separation of the *Leguminosae* family.

Polyols, which can act as osmoprotective solutes and antioxidants, have been detected in both sink and source tissues in plants. The expression of different *PLT* genes could be detected in sinks and/or source tissues. Analysis showed that most of *LjPLTs* genes were expressed to varying levels in different tissues (Fig 4). Clade I genes were expressed in all organs tested, and their transcripts accumulated to an especially high degree in chlorenchyma tissues. The *LjPLT12* gene of clade III was expressed mainly in pods and seeds, whereas the *LjPLT8* gene was expressed weakly in these tissues (Fig 4). Four genes (*LjPLT3*, *LjPLT4*, *LjPLT11* and *LjPLT14*) of clade II were very highly expressed in nodules. The divergent expression patterns of the *PLT* genes suggested that subfunctionalization has occurred during the evolutionary process in *L*. *japonicus*.

As a nitrogen-fixing legume, *L*. *japonicus* develops root nodules that harbor *Rhizobium* bacteria. The polyols including mannitol and sorbitol, were found to be accumulated in nodules in *L*.*japonicus* and osmotic stress is a normal aspect of nodule physiology [22], which may indicate polyols could as osmoprotectants in nodules. On the other hand, a proteomic study identified a putative mannitol transporter on isolated peribacteroid membrane/SM [23], which suggests that polyols may be transported between the plant and bacteroids. Therefore, *LjPLT3*, *LjPLT4*, *LjPLT11* and *LjPLT14* with high expression level in nodules could be involved in osmotic stress in nodule development and also in transportation of polyols between plants and rhizobia (Fig 4). The expression of three *LjPLT* genes induced by inoculating with *M*. *loti* (Fig 5) indicated that polyol may as antioxidants in plant oxidative burst response to Rhizobia triggered in the *L*. *japonicus-Rhizobium* interaction.

*PLTs* belong to the monosaccharide transporter (MST) gene family [40, 41]. MSTs have been found to be localized on the PM, plastid envelope [42, 43], tonoplast [44] or Golgi apparatus [45, 46]. Previous studies indicated that PLT proteins were localized to the PM and they have been characterized as being xylitol specific H^+^-symporters [24]. All of the PLT proteins studied in different plant species belong to clade I (Fig 2). Similarly, in this study, we inferred that three LjPLT proteins of clade I may be PM-localized by carrying out transient expression analyses of GFP-LjPLT constructs in Arabidopsis protoplasts (Fig 7A). However, five LjPLTs from the other three clades were localized mainly on the Golgi apparatus (Fig 8A and 8B). Although the transport activities of the other LjPLT proteins have not been characterized, we speculate that they are polyol and/or monosaccharide transporters based on their sequence characteristics. The fact that LjPLTs in the clade I are localized to the PM suggests that they may function in the cell-to-cell distribution of polyol and/or monosaccharide in *L*. *japonicus*. The localization of LjPLTs in the Golgi apparatus suggested a possible role for these PLT proteins in the distribution of polyol and/or monosaccharide between the Golgi apparatus and cytosol (Fig 8). Plasma membrane-localized proteins, such as transporters, are synthesized, folded and assembled in the endoplasmic reticulum and subsequently transported to the cell surface through the secretory pathway. The conventional secretory path is through the Golgi apparatus and the trans-Golgi network [47]. In a few cells (about 10–20%), clades II, III and IV LjPLT proteins were also located at the PM (Fig 8), which implied that these PLTs may be trafficked from Golgi apparatus to PM and function in the cell-to-cell distribution of polyol and/or monosaccharide. The assignment of a transmembrane protein to the PM is dynamically regulated by these intracellular trafficking processes in response to intracellular and extracellular cues [48]. Further investigations into the functions of *PLT* genes in the legume-*Rhizobium* interaction are in progress.

## Supporting information

S1 Fig Phylogenetic tree of polyol transporters in *Leguminosae*. (TIF)

S1 Table Primers used in our study. (XLSX)

S2 Table The *LjPLT* genes identified in this study. (XLSX)

S3 Table Gene ID and accession numbers for the *PLT* genes analyzed. (XLSX)

S4 Table Known *cis*-acting elements in the 2000 bp fragment from ATG of *LjPLT* promoters. (XLSX)
