# Supplementary figures and images for: Heterogeneity in the expression and subcellular localization of POLYOL/MONOSACCHARIDE TRANSPORTER genes in Lotus japonicus
Source: PLoS One. 2017 Sep 20;12(9):e0185269. doi: 10.1371/journal.pone.0185269 (PMC5607196; doi:10.1371/journal.pone.0185269)

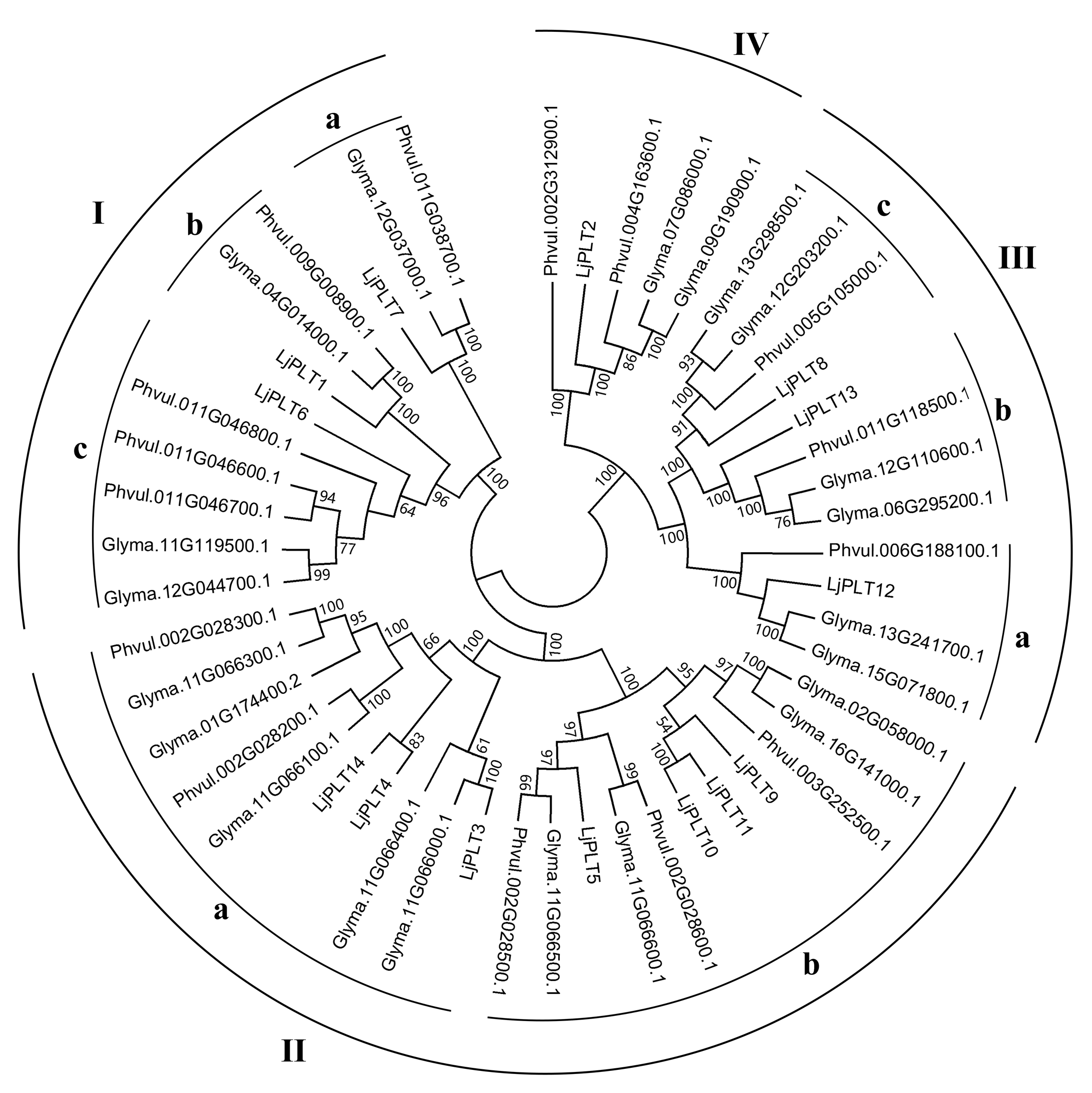

Supplement: S1 Fig — (TIF) [file pone.0185269.s001.tif]
